# Biocompatibility of Nanocellulose-Reinforced PVA Hydrogel with Human Corneal Epithelial Cells for Ophthalmic Applications

**DOI:** 10.3390/jfb10030035

**Published:** 2019-08-01

**Authors:** Gopi Krishna Tummala, Viviana R. Lopes, Albert Mihranyan, Natalia Ferraz

**Affiliations:** Nanotechnology and Functional Materials, Department of Engineering Sciences, Uppsala University, Box 534, 751 21 Uppsala, Sweden

**Keywords:** cellulose nanocrystals, cornea regeneration, contact lens, poly(vinyl alcohol), therapeutic lens

## Abstract

Transparent composite hydrogel in the form of a contact lens made from poly(vinyl alcohol) (PVA) and cellulose nanocrystals (CNCs) was subjected to in vitro biocompatibility evaluation with human corneal epithelial cells (HCE-2 cells). The cell response to direct contact with the hydrogels was investigated by placing the samples on top of confluent cell layers and evaluating cell viability, morphology, and cell layer integrity subsequent to 24 h culture and removal of the hydrogels. To further characterize the lens–cell interactions, HCE-2 cells were seeded on the hydrogels, with and without simulated tear fluid (STF) pre-conditioning, and cell viability and morphology were evaluated. Furthermore, protein adsorption on the hydrogel surface was investigated by incubating the materials with STF, followed by protein elution and quantification. The hydrogel material was found to have affinity towards protein adsorption, most probably due to the interactions between the positively charged lysozyme and the negatively charged CNCs embedded in the PVA matrix. The direct contact experiment demonstrated that the physical presence of the lenses did not affect corneal epithelial cell monolayers in terms of integrity nor cell metabolic activity. Moreover, it was found that viable corneal cells adhered to the hydrogel, showing the typical morphology of epithelial cells and that such response was not influenced by the STF pre-conditioning of the hydrogel surface. The results of the study confirm that PVA-CNC hydrogel is a promising ophthalmic biomaterial, motivating future in vitro and in vivo biocompatibility studies.

## 1. Introduction

Cornea is the transparent tissue that, together with sclera, forms the anterior surface of the human eye. The ability to see properly depends on the optical properties of the cornea, since two-thirds of the refractive power of the eye is due to the cornea while the remaining one-third is due to the eye lens [1]. The tear film has an important role in enabling optical functions of the cornea by forming a thin smooth liquid surface, which minimizes undesired surface scattering. Tear fluid not only cleans the conjunctiva and eye from time to time, but also contains components of the immune system like immunoglobulins, lysozyme, polymorphonuclear leukocytes, etc. [2,3]. Any damage to the cornea due to accident or infection leads to impaired vision and ultimately blindness. It is estimated that about 23 million people worldwide are corneal blind at least in one eye [4].

The only major curative treatment available for corneal blindness is cornea transplant surgery. Though the risk of rejection is low due to the avascular nature of the cornea [5], the biggest problem is the shortage of donor corneas and the logistics associated with it. The alternatives to allogenic cornea implants are keratoprosthetics (or synthetic corneas) [6] and bioengineered cornea regeneration implants containing stem cells [6,7,8,9,10,11]. Because of the complex surgery procedure and post-operative complications, synthetic corneas are generally considered for implantation only in cases of repeated rejection of allogeneic tissue by the host [12]. 

Several polymers have been studied to develop biomimetic cornea regeneration implants [5]. A synthetic cornea needs to suffice several parameters, i.e., protection, transparency, and refractive power [13]. Due to the structural similarities of extracellular matrix and the three-dimensional framework supporting cell proliferation and survival, hydrogels have unique desirable properties for cornea regeneration [14]. Cornea regeneration biomaterials, including recombinant human collagen-based, fibrin-based, silk-based, and self-assembled corneal implants, have been previously described in the literature [15]. 

Another major group of ophthalmic disorders includes ophthalmic refractive errors, such as myopia, hyperopia, and keratoconus. Various hydrogels in the form of soft contact lenses are commonly used for refractive correction [14,16]. The Food and Drug Administration (FDA) classifies soft contact lenses in four major groups, depending on their water content (high vs. low) and type of polymer (non-ionic vs. ionic) [17,18,19]. Groups 1 and 2 comprise non-ionic polymers with low and high water content, respectively. Similarly, groups 3 and 4 include ionic polymers with low and high water content, respectively. 

In general, irrespective of their final use, biomimetic hydrogels for ophthalmic applications are highly demanded. Several factors require consideration when designing novel hydrogels for ophthalmic applications. Physical structure, surface morphology, elasticity, water content, and oxygen permeability are important properties, which determine the applicability of the hydrogel. Besides, the characteristics of a biocompatible material depend on the foreseen use—a contact lens material should have a minimum effect on the normal hemostasis of the corneal epithelium [20], i.e., a so-called bioinert material, while a biomaterial for cornea regeneration should be bioactive, supporting re-epithelialization and re-innervation after implantation into a host cornea [21]. A better understanding of the cellular basis of ophthalmic material´s biocompatibility could contribute to the design of improved materials toward specific applications. 

Poly(vinyl alcohol) (PVA) is one synthetic polymer which has a long track record in ophthalmic care [22]. Early work by Hyon et al. [23,24] and Kita et al. [25] described the development of PVA hydrogel-based soft contact lenses. Lately, authors have investigated the use of PVA-based hydrogels as platforms to deliver bioactive compounds [26,27,28,29]. Besides, the use of PVA-based materials to prepare artificial cornea has also been proposed [30,31,32]. PVA hydrogels can be produced by physical, e.g., cryogelation [33], chemical, e.g., glutaraldehyde [34], or radiation, e.g., UV- or γ-ray crosslinking [35]. PVA hydrogels are elastic, have a low coefficient of friction, and show mechanical behavior similar to collagen [36,37]. 

We have previously reported the synthesis process of cellulose nanocrystal (CNC)-reinforced PVA hydrogel following a physical route of crosslinking by cryogelation from a mixed DMSO/water solvent system [38,39]. The produced hydrogel shows exceptionally high water content [39], high transparency and low light scattering [38], as well as biomimetic collagen-like mechanical properties [37]. Furthermore, PVA-CNC lenses are a promising platform for enzymatically-triggered ophthalmic drug delivery [40]. In the present work we investigate the biocompatibility of PVA-CNC hydrogel with corneal epithelial cells as a step forward in the material´s characterization towards clinical ophthalmic applications. Two different types of biocompatibility tests are selected having in mind the potential uses of the CNC-PVA hydrogels (i.e., as contact lenses and as material for corneal regeneration). Besides, protein adsorption on the hydrogel surface is investigated and the effect of preconditioning the hydrogel surface with simulated tear fluid (STF) on cell response is also evaluated. 

## 2. Results and Discussion

### 2.1. Material Characterization

**Figure 1:**
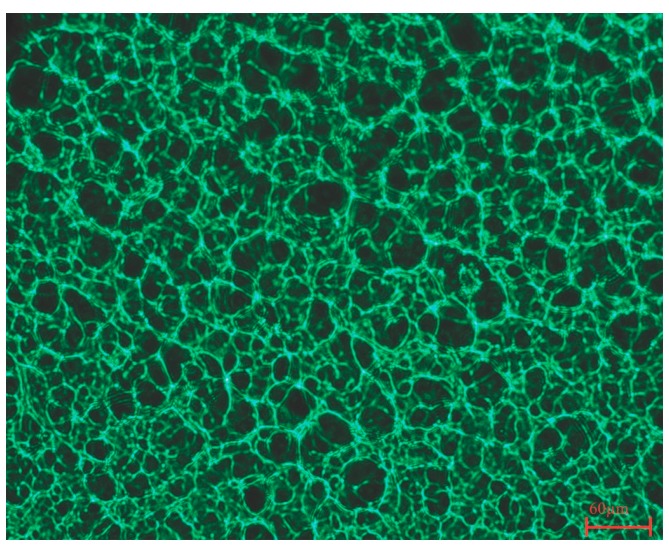
Cross section image of cellulose nanocrystals (CNC)-poly(vinyl alcohol) (PVA) hydrogel acquired with an IF 550 green contrast color filter. Scale bar corresponds to 60 µm.

The CNC-reinforced PVA hydrogel under study has a 3D network architecture as depicted in Figure 1. The pore size is estimated to be in the range between 10 and 30 µm. The morphology of the contact lenses is covered in detail in a previous publication [38].

Protein adsorption on the PVA hydrogels and on the reference material Nelfilcon A was investigated by incubating the lenses with STF for 24 h, after which the adsorbed proteins were eluted and quantified. The results showed that the CNC-PVA lens has protein adsorption of 127 ± 37 µg/lens, whereas commercially modified PVA hydrogel, i.e., Nelfilcon A, has protein adsorption of 13 ± 4 µg/lens. It is evident that the CNC-PVA lens is more prone to adsorb proteins compared to Nelfilcon A. Nelfilcon A is a non-ionic hydrogel lens with high water content (FDA group 2 lens), and, therefore, low protein adsorption was expected. It was shown earlier that contact lenses made from ionic polymers with high water content (FDA group 4 lenses) present high levels of non-specific protein adsorption [41]. In the case of the CNC-PVA hydrogel, the presence of negatively charged CNCs embedded in the PVA polymer matrix could be responsible for large adsorption of the positively charged lysozyme by the lens. 

It should be mentioned that the tear fluid consists of more than 400 different proteins, with a wide range of molecular weight and isoelectric points [42]. The STF used in this study contained only lysozyme, albumin, and gamma-globulin. Thus, the formation of a protein layer in vivo would be a much complex phenomenon than the in vitro adsorption assay performed here, e.g., competition between a large number of different proteins for the available material surface may influence the composition of the final protein layer. Besides, changes in protein conformation and denaturation during the adsorption process should also be considered when evaluating the protein–lens interactions since such changes could influence the risk of bacterial infection and inflammatory reactions [43].

### 2.2. Biocompatibility Studies

To study material–cell interactions, designing in vitro assays that take into consideration the material’s specific application permits addressing potential biocompatibility challenges at early stages of product development. Therefore, we selected two different types of biocompatibility tests having in mind the potential use of the CNC-PVA hydrogels as contact lenses and as material for corneal regeneration. These studies complement our previous work where we did a first screening of the PVA-CNC cytocompatibility by investigating toxic leachables (i.e., indirect cytotoxicity tests) and by performing microscopy evaluation of green-fluorescence protein (GFP)-labeled human corneal epithelial cells cultured on the surface of the hydrogels (i.e., qualitative evaluation of cell response to the hydrogels) [44].

**Figure 2:**
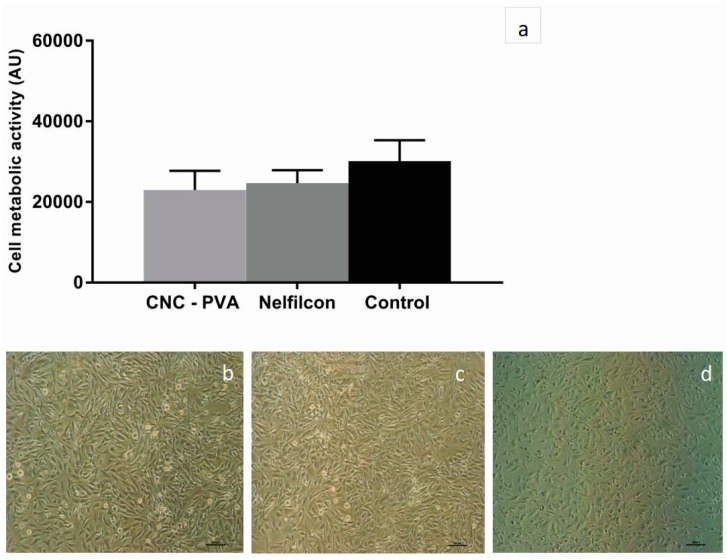
Human corneal epithelial cells (HCE-2 cells) after 24 h direct contact with the hydrogels (CNC-PVA and Nelfilcon) and subsequent removal of the materials. (**a**) Cell metabolic activity assessed by the Alamar blue assay. Data represent the mean ± standard error of the mean (SEM) for n = 3. One way ANOVA was applied. No significant difference was observed between cells exposed to the hydrogels and the control (untreated cells); (**b**–**d**) representative light microscopy images of the cell layers after direct contact with (**b**) CNC-PVA and (**c**) Nelfilcon A; while (**d**) corresponds to untreated cells. The scale bar corresponds to 100 µm.

In the present work, the response of human corneal epithelial cells (HCE-2) to direct contact with the hydrogels was evaluated by placing the samples on top of confluent cell layers and subsequent 24 h culture. Thereafter, the hydrogels were carefully removed, and cell viability and morphology were evaluated. Results indicated that cell viability was not affected by the presence of the CNC-PVA lenses, since no significant difference in cell metabolic activity was seen between cells in contact with the material and the untreated cells, as shown in Figure 2a. Besides, light microscopy images of the cell layer after contact with the hydrogels revealed that the cell morphology was maintained, and that the integrity of the cell layer was not affected by the presence of the CNC-PVA lenses, as shown in Figure 2b. Similar results were obtained with the reference material Nelfilcon A, as shown in Figure 2a,c. These results showed that the physical presence of the lenses does not affect corneal epithelial cell monolayers and could be an indication that the novel PVA lenses would not disturb the corneal hemostasis, at least in terms of cell proliferation [20]. Thus, the composite hydrogel fulfills one of the biocompatibility aspects of materials for contact lens applications. 

**Figure 3:**
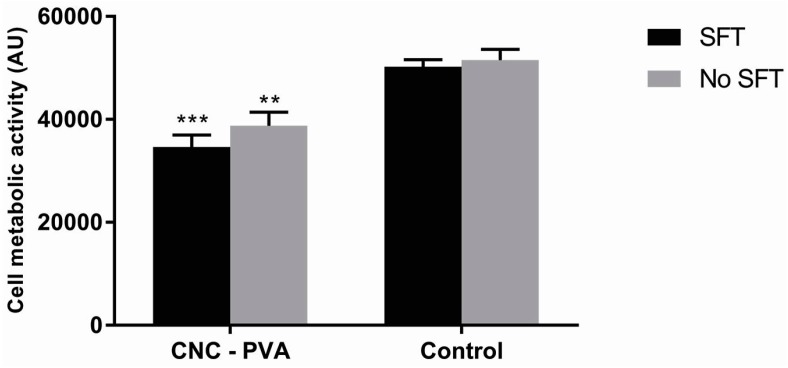
Cell viability of HCE-2 cells cultured on CNC-PVA lens and tissue culture plate (control) for 24 h, with and without simulated tear fluid (STF) pre-conditioning of the material surfaces (SFT and no SFT, respectively). Data represent the mean ± SEM for n = 3. Statistically significant differences between the CNC-PVA and the controls are indicated with ** *p* < 0.01 and *** *p* < 0.001 by two-way ANOVA.

**Figure 4:**
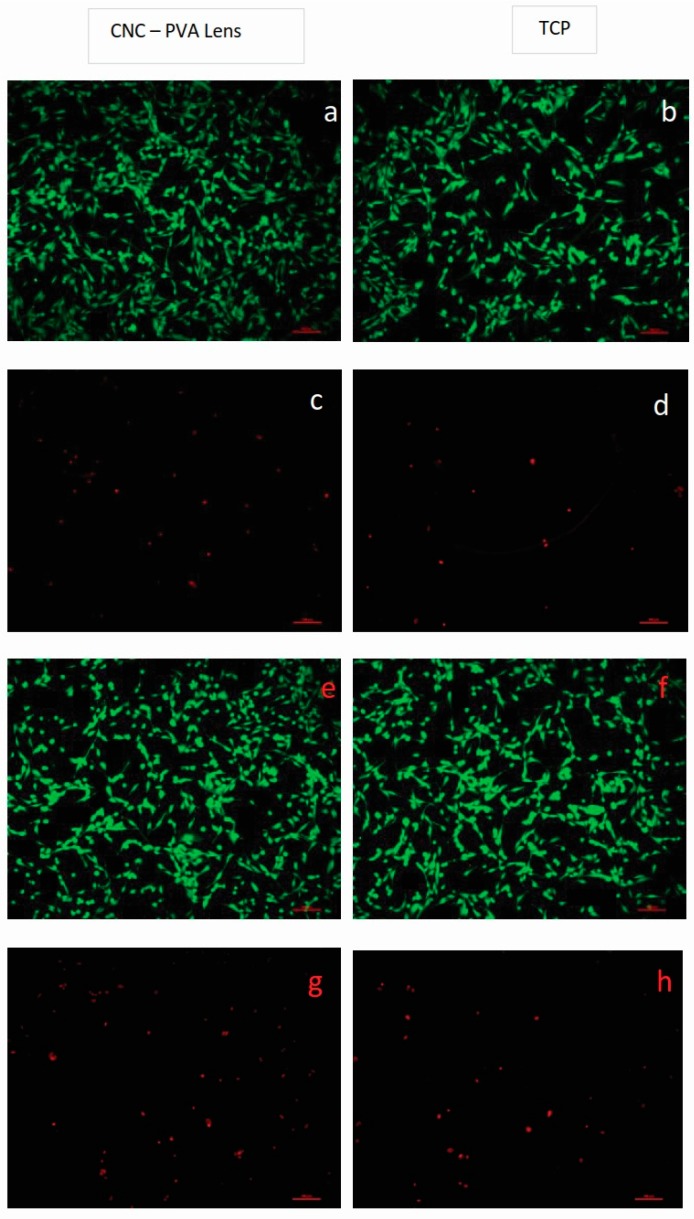
Live/dead staining of HCE-2 cells cultured on CNC-PVA lens and tissue culture plate (TCP) for 24 h. Panels (**a**,**b**) and (**c**,**d**) show images of live (green) and dead cells (red), respectively, in surfaces pre-conditioned with STF, while panels (**e**,**f**) and (**g**,**h**) show images of live (green) and dead cells (red), respectively, in materials not previously exposed to STF. The scale bar corresponds to 100 µm.

To further characterize the lens–cell interactions and having in mind the potential application of the material in cornea regeneration, HCE-2 cells were seeded on the material and cell viability and morphology were evaluated after 24 h culture. The experiments were carried out with and without STF pre-incubation of the lenses. Cell viability results, measured as cell metabolic activity, are depicted in Figure 3, while fluorescence microscopy images of live/dead stained cells are shown in Figure 4. Results indicate that the CNC-PVA hydrogels support corneal epithelial cell attachment, with cells displaying the typical morphology of the epithelial cells and few numbers of non-viable cells (i.e., cells with compromised membrane integrity), comparable to the cells cultured on the tissue culture material, as shown in Figure 4. When quantifying the number of metabolically active cells on the surface of the lenses, results indicated a significant difference compared to the control, as shown in Figure 3. However, such differences represent only 30%, thus relatively good attachment and metabolic activity of the cells on the studied materials can be claimed.

As mentioned before, the formation of tear film on the surface of the ophthalmic material is expected upon contact between the material and tears. The presence of the protein film is anticipated to influence the interactions at the material surface, including those with corneal epithelial cells [2]. Thus, we investigated the effect of STF pre-conditioning of the lens surface on cell attachment and viability, so as to mimic the eye environment. However, no significant differences between the STF and no STF experiments were found. Even if we detected relatively high protein adsorption, it seems that the adsorption of lysozyme and gamma-globulin on the CNC-PVA hydrogel was such that it did not promote changes in cell response compared with the uncoated material. 

The results presented here are a first indication of the material´s good biocompatibility towards corneal epithelial cells. Together with our previous findings of ex vivo suturing in porcine cornea [44], these results push forward the investigation on the potential applications of the CNC-PVA hydrogels in cornea regeneration. In this sense, future studies will focus on long-term cell proliferation, studies on the functionality of the cell layer, e.g., presence of tight junctions, and pre-clinical in vivo studies in animals.

## 3. Materials and Methods

### 3.1. Materials

Microcrystalline cellulose, Avicel PH 101, (MCC), 2,2,6,6-Tetramethylpiperidine-1oxyl radical (TEMPO), NaBr, NaCl, NaClO, NaOH, PVA (average Mw 146,000–186,000 and degree of saponification 99.9%), dimethyl sulfoxide (DMSO), aqueous hydroxyl amine (50%), sodium dodecyl sulfate (SDS), ethanol, hydrochloric acid, acetic acid, iso-propanol, Dulbecco’s phosphate buffered saline (PBS), lysozyme, gamma-globulin, bovine serum albumin, and a live-dead staining kit were purchased from Sigma Aldrich (Stockholm, Sweden). A micro bicinchoninic acid (BCA) protein assay kit was purchased from Thermo Fisher Scientific (Waltham, MA, USA). Keratinocyte serum free medium (KSFM) and its supplements were purchased from Life Technologies (Bleiswijk, the Netherlands). Alamar blue (AB) cell viability reagent was purchased from Thermo Fisher Scientific (Waltham, MA, USA). All the chemicals used were of reagent or analytical grade. 

### 3.2. TEMPO-Mediated Oxidation of Cellulose

To produce carboxylated CNC, MCC was oxidized according to a previously verified method [45]. In short, 5 g MCC were dispersed in 400 mL of deionized water and 90 mg of TEMPO and 1 g of NaBr dissolved in 100 mL deionized water were added to it. The mixture was kept stirred at 500 rpm. To the mixture, 10 mL of 10% (w/w) NaClO (pH 11, adjusted with 1M HCl) were added at intervals of 30 min for 3.5 h. The pH of the system was maintained at 10.5 for the entire period of reaction by adding 1 M NaOH. The reaction was quenched after 3.5 h by adding 10 mL of ethanol. Cellulose was washed three times with deionized water by centrifugation and then dialyzed for three days. Cellulose was then collected by centrifugation and dispersed in deionized water by ultrasonication (Vibracell 700 W, 20 KHz, Sonics, Newtown, CT, USA) for 5 min to obtain a transparent gel.

### 3.3. Preparation of CNC-Reinforced PVA Hydrogel

Crosslinking by cryogelation from mixed DMSO/water solvent system was the chosen method to produce the PVA hydrogel. The selection of the mixed solvent system ratio was based on previous works where the effect of different DMSO/water ratios on the hydrogel’s transparency and mechanical strength was investigated [23,24]. Briefly, 3 g of PVA were added to a mixed solvent system of DMSO and deionized water (80:20) so as to obtain a concentration of 10% (w/w). The PVA solution was obtained by heating the mixture at 100 °C under agitation in an oil bath for about 2 h. CNC (1% w/w of PVA) was added to the PVA solution and further mixed for an hour. The homogenous and transparent solution obtained by this method was cast in molds and allowed to gel at −20 °C for 24 h. The formed gels were collected from the molds, and the DMSO in them was exchanged with deionized water by dialyzing the gels in excess water for at least 48 h. The obtained hydrogel lenses have a diameter of 10 mm and thickness of 500 µm and were stored in deionized water. Previous to the biocompatibility studies, the CNC-PVA lenses were sterilized by UV radiation. 

### 3.4. Optical Microscopy

Cross-section images of the composite hydrogel were acquired with an Olympus AX70 microscope (Olympus, Tokyo, Japan). Disc shaped, 12.5 mm thick hydrogel samples were used. The microscope mounted with a CCD (couple-charged device) camera and a DeltaPix software program (DeltaPix, Smorum, Denmark) was used to acquire the pictures. IF 550 green contrast color filter was used for better visualization.

### 3.5. Protein Adsorption Assay

STF consisting of lysozyme (2.68 mg/mL, MW 14 KDa, isoelectric point (IP) 11.4), gamma-globulin (1.34 mg/mL, MW > 180 KDa, IP 6.85–6.95), bovine serum albumin (2.68 mg/mL, MW 66 KDa, IP 4.7–5.3), sodium chloride (6.50 mg/mL), D-glucose (6.50 mg/mL), and calcium chloride dihydrate (0.08 mg/mL) was prepared in deionized water and the pH adjusted to 7.4 using 0.01 M HCl [46]. The solution was mixed for 30 min using a magnetic stirrer and filtered using an 80 µm filter. CNC-PVA lenses and samples of the commercial modified PVA-based contact lens Nelfilcon A were immersed in 2 mL of STF and incubated for 24 h followed by three washing steps with PBS. The lenses were later placed in 2% (v/v) SDS in deionized water for 2 h to elute the adsorbed proteins. Eluted proteins were quantified using a BCA assay (Micro BCA™ Protein assay kit, Pierce; Thermo Fisher Scientific, Waltham, MA, USA). Results are expressed as µg/lens. 

### 3.6. Biocompatibility Studies

#### 3.6.1. Cell Culture

Human corneal epithelial cells (HCE-2) were purchased from American Type Culture Collection (ATCC). HCE-2 cells were cultured in KSFM supplemented with 0.05 mg/mL bovine pituitary extract, 5 ng/mL epidermal growth factor, 500 ng/mL hydrocortisone, and 5 ng/mL insulin. Cells were cultured in a humidified atmosphere at 37 °C, 5% CO_2_, and sub-cultured at 70–80% confluency. Cells were harvested using trypsin treatment and counted using a hemocytometer. Cell viability was assessed using trypan blue staining.

#### 3.6.2. Direct Cytocompatibility Test

HCE-2 cell suspensions were prepared in complete KSFM at a density of 1 × 10^5^ cells/mL and seeded on a 24-well tissue culture plate (5 × 10^4^ cells per well). Cells were then incubated for 24 ± 2 h at 37 °C, 5% CO_2_ in a humidified atmosphere before applying the lens samples on top of the confluent cell layer. Nelfilcon A lenses were used as reference. Cells cultured on a tissue culture plate (TCP) without the presence of the lenses served as a control (untreated cells). The cells were incubated for further 24 ± 2 h at 37 °C, 5% CO_2_ in a humidified atmosphere and thereafter the samples were carefully removed. Cell morphology and the integrity of the cell layers were analyzed by light microscopy (Nikon Eclipse TE2000, Tokyo, Japan), while cell metabolic activity was assessed by the AB assay. In this assay, 500 μL of AB reagent diluted 1:10 in PBS was added to the wells and incubated for 90 min at 37 °C, 5% CO_2_ in a humidified atmosphere. Aliquots of 100 μL from each well were transferred to a black 96-well plate and fluorescence intensity was measured at 560 nm excitation wavelength and 590 nm emission wavelength using a spectrofluorometer (Tecan infinite^®^ M2000, Männedorf, Switzerland). Samples were run in triplicate and the experiment was repeated three times.

#### 3.6.3. Cell Adhesion and Viability

Lens samples with and without STF pre-conditioning were placed in 24-well tissue culture plates and 5 × 10^4^ HCE-2 cells suspended in complete KSFM were seeded on top of the CNC-PVA lenses (500 μL cell suspension per well) and cultured for 24 ± 2 h at 37 °C, 5% CO_2_ in a humidified atmosphere. Cells cultured on TCP served as a control. Samples were run in triplicate and the experiment was repeated three times.

- Live-dead staining: Adherent cells were double stained with calcein-AM and propidium iodine (PI) to visualize live and non-viable cells, respectively, after 24 h culture. Cell culture medium was removed from the wells, the lenses were carefully rinsed with PBS, and 300 µL of assay solution (2 μL calcein-AM and 1 μL PI per mL of PBS) was added to each sample and incubated for 15 min at 37 °C, 5% CO_2_. The stained cells were imaged using a fluorescence microscope (Nikon ECLIPSE TE2000-U, Tokyo, Japan) with λ_ex_ 490 nm, λ_em_ 515 nm filter to visualize live cells and λ_ex_ 535 nm, λ_em_ 617 nm filter to visualize cells with compromised membrane integrity.
- Cell viability test: Cell metabolic activity, an indicator of cell viability, was assessed by the AB assay following the protocol described in Section 3.6.2.

### 3.7. Statistical Analysis

All data were expressed as the mean ± standard error of the mean (SEM) of three independent experiments. One-way and two-way ANOVA followed by Sidak’s multiple comparison post-hoc tests were performed using GraphPad Prism version 7.00 for Windows (GraphPad Software, La Jolla, CA, USA, www.graphpad.com); *p* values < 0.05 were considered statistically significant. 

## 4. Conclusions

In the present study we found that CNC-reinforced PVA hydrogel of high water content has macroporous 3D network architecture with pore size ranging from 10 to 30 µm. The surface of the hydrogel material was found to have affinity towards protein adsorption. The hydrogel did not affect corneal epithelial cell monolayer cultures when in direct contact, demonstrating the non-toxic and cytocompatible profile of the CNC-PVA hydrogel. Furthermore, it was found that viable corneal cells adhered to the hydrogel and that such response was not influenced by the STF pre-conditioning of the hydrogel surface. Overall, the PVA-CNC hydrogel shows potential for continued pre-clinical studies for ophthalmic applications.
